# Dietary *Yucca schidigera* Extract Supplementation During Late Gestating and Lactating Sows Improves Animal Performance, Nutrient Digestibility, and Manure Ammonia Emission

**DOI:** 10.3389/fvets.2021.676324

**Published:** 2021-07-21

**Authors:** Fang Chen, Yantao Lv, Pengwei Zhu, Chang Cui, Caichi Wu, Jun Chen, Shihai Zhang, Wutai Guan

**Affiliations:** ^1^Department of Animal Science, South China Agricultural University, Guangzhou, China; ^2^National Engineering Research Center for Breeding Swine Industry, Guangzhou, China; ^3^College of Animal Science and Technology, Zhongkai University of Agriculture and Engineering, Guangzhou, China; ^4^Innovative Institute of Animal Healthy Breeding, College of Animal Sciences and Technology, Zhongkai University of Agriculture and Engineering, Guangzhou, China

**Keywords:** animal performance, antioxidative, manure, nitrogen loss, sow, *Yucca schidigera* extract

## Abstract

This study was conducted to investigate the effect of dietary *Yucca schidigera* extract (YSE) supplementation to sow performance, nutrients digestibility and ammonia emission of manure. Total 80 sows were randomly divided into 4 groups and fed with either control, control + 0.06% YSE, control + 0.12% YSE or control + 0.24% YSE diet from day 80 of gestation to day 21 of lactation. The results showed that dietary YSE supplementation resulted in trends toward a reduced number of stillbirth piglets (*P* = 0.08), weak piglets *(P* = 0.06), pre-weanling mortality (*P* = 0.04) and diarrhea (*P* = 0.03), and improved apparent digestibility of dry matter (*P* = 0.04). Besides, YSE supplementation significantly increased catalase activity (*P* = 0.02) while decreasing malonaldehyde levels (*P* = 0.04) in sow blood. Furthermore, the loss of total nitrogen, urea nitrogen and ammonia nitrogen in sow manure were significantly reduced with supplementation of YSE. In summary, supplementation of YSE in sow diet during late gestation and lactation could improve sow and litter performance, nutrient digestibility, and reduce nitrogen loss in sow manure during storage.

## Introduction

Ammonia emissions from animal facilities are frequently the subject of odor complaints and public concern due to the possibility of their negative health effects in both humans and animals. Livestock and poultry may develop a variety of disorders when exposed to high levels of ammonia for extended periods of time (1, 2). Therefore, it is very important to develop efficient strategies to reduce NH_3_ emissions from livestock manure.

*Yucca schidigera*, a species of the yucca plant that is native to the southwestern United States and Mexico, is rich in natural steroid saponins, numerous enzymes, antioxidants and resveratrol (3). In recent times, yucca plant extracts have been widely used as natural additives in the food and livestock industries (4). *Yucca schidigera* extract (YSE) has been shown to not only improve livestock and poultry performance, including weight gain, feed efficiency and health (5–7), but also reduce ammonia emissions from animal manure and consequently control odor in animal facilities as well (8–10).

Although previous studies have been conducted to investigate the effect of dietary YSE supplementation on performance and NH_3_ emission in pig industry, most of these studies focused on growing pigs rather than sows which produce more manure than young pigs. Therefore, different levels of YSE were supplemented into sow diets during late gestation and lactation to evaluate their effects on animal performance, nutrient digestibility, blood gas parameters, and oxidative status in present study. Furthermore, effect of YSE on total nitrogen, ammonia nitrogen and urease activity in sow manure during storage with time passage were also determined to investigate the effect of YSE to ammonia emission.

## Materials and Methods

The experiment was carried out according to Chinese guidelines for animal welfare and the National Institutes of Health guide for the care and use of Laboratory animals. All the experimental procedures performed in this study were approved by the South China Agricultural University Animal Care and Use Committee.

### Animals and Diets

The YSE used in this study was a commercial product provided by Shanghai Hengtai Company, and it contained >10.5% saponin and >318 mg/kg resveratrol. A total of 80 sows (Large White, four to six parities) from the Huizhou Swine Breeding Center were used in this study, and they were housed in individual feeding stalls with free access to water during the entire trial. Sows were allotted to 4 treatment groups with 20 sows per treatment, and they were fed diets containing either 0.0% (control), 0.06, 0.12, and 0.24% YSE from day 80 of gestation through day 21 of lactation. The composition of the experimental diets and their nutrient levels are described in Table 1. The nutrient levels of the diets met or exceeded the requirements for sows during late gestation and lactation. The sows were kept in single crates (0.6 × 2.0 m) from insemination to d 110 of gestation, at which time they were transported to the farrowing facility where they were placed in individual farrowing crates (2.4 × 2.4 m). All sows were managed in the gestation facility from day 80 to day 109 of gestation and then transferred to lactation facilities to prepare for farrowing. Feed consumption was limited to 2.5 kg/d during gestation and was then increased progressively by 0.5 kg/d during lactation. Within 48 h postpartum, litter size was standardized to 9 ± 1 piglets per litter by cross-fostering within the same treatment (a total of 200 piglets per treatment), and standard practices were applied as follows: iron injection (200 mg Fe as gleptoferron), needle teeth and tail clipping. The piglets were weaned at d 21 of lactation.

**Table 1A:** Composition and nutritional value of the diet (as-fed basis).

**Item**	**Gestation**	**Lactation**
**Ingredient (%)**		
Corn	66.00	61.00
Soybean meal	20.00	25.00
Bran	10.00	6.00
Fish meal (62.8%)	–	2.00
Soybean oil	–	2.00
Premix^a^	4.00	4.00
Total	100.00	100.00

a *Vitamin and mineral premix supplied per kilogram of complete diet: 100 g NaCl, 50 mg Zn (ZnSO4·H_2_O), 80 mg Fe (FeSO4·H_2_O), 20 mg Mn (MnSO4·H_2_O), 5 mg Cu (CuSO4·5H2O), 0.14 mg I (CaI2O6), 0.3 mg Se (Na2SeO3), 13,000 IU vitamin A, 4000 IU vitamin D3, 30 IU or 90 IU vitamin E, 4 mg vitamin K3, 4 mg vitamin B1, 10 mg vitamin B2, 4.8 mg vitamin B6, 0.034 mg vitamin B12, 40 mg niacin, 20 mg d-pantothenate, 2 mg folic acid, and 0.16 mg d-biotin*.

**Table 1B:** Composition and nutritional value of the diet (as-fed basis).

**Nutritional level^a^**	**Gestation**	**Lactation**
Apparent digestibility (MJ/kg)	13.21	13.90
Crude protein	15.51	17.96
Crude fat	3.12	4.89
Dietary fiber	3.86	3.39
Ash	5.16	5.09
Ca	0.86	0.86
Total P	0.52	0.57
Lysine	0.78	0.95
Methionine + cystine	0.55	0.60
Threonine	0.66	0.71
Tryptophan	0.21	0.23

a *Nutritional levels were analyzed value except calculated apparent digestibility*.

### Sample and Data Collection

#### Sow and Litter Performance

Once farrowing was completed, the number of piglets born (total, live, stillborn, mummy), litter birth weights, and individual piglet weights were recorded. At days 2, 7, 14, and 21 of lactation, litter size and litter weights were recorded, as well as the feed intake of each lactating sow and oestrus rate at day 5 and 7 after weanling.

#### Blood Sampling

At day 100 of gestation and day 15 of lactation, blood samples (20 mL per sow) from 6 sows per treatment were taken by ear venipuncture using heparinized vacutainer tubes (10 mL) and normal tubes (10 mL). Plasma was harvested after centrifugation at 3,000 × g for 10 min for subsequent blood gas analysis, and serum was harvested to assess antioxidative capacity.

#### Feces Sampling for Nutrient Digestibility

Fresh fecal samples were collected quantitatively twice daily from each sow from day 93 to day 95 of gestation and from day 14 to day 16 of lactation, and they were stored at −20°C immediately after collection. Ash insoluble in hydrochloric acid was used as an indigestible marker to calculate the apparent nutrients total tract digestibility (11). Fecal samples were dried in a 50°C forced-air drying oven and then ground to pass through a 1 mm screen before chemical analysis.

#### Feces and Urine Sampling for Nitrogen Related Parameters

Fresh feces and urine samples were collected from sows on day 100 of pregnancy and day 15 of lactation and 10% hydrochloric acid was added immediately to fix nitrogen. Feces and urine samples from individual sows were collected separately in clean plastic bags to ensure that there was no mixing prior to the ensuing experiments. Both the feces and urine samples were taken directly upon excretion from the sows to prevent any contact with the barn floor. All samples were kept at 4°C during transportation and then stored at −80°C until analysis.

#### Colostrum and Milk Sampling

The cololstrum and milk samples were sampled after intramuscular injection of 20 IU oxytocin within 24 h after parturition, on day 14 and day 21 of lactation.The samples were immediately frozen and stored at −80°C until analysis.

### Chemical Analysis

#### Diet and Feces Composition

The dry matter, crude protein, crude fiber, starch, phosphorus, calcium, copper, iron, zinc and manganese content of the diets were assessed according to the Chinese standard methods (GB/T 6435–2014, GB/T 6432-1994, GB/T 6434-2006, GB/T 6433-2006, GB/T 20194-2006, GB/T 6437-2002 and GB/T 13885-2017).

#### Antioxidant Status

The antioxidant status, including total antioxidant capacity (T-AOC), superoxide dismutase (SOD), glutathione peroxidase (GSH-Px), glutathione (GSH), catalase (CAT) and malonaldehyde (MDA) levels in plasma, colostrum and milk were measured as described in our previous studies (12), using commercially available kits (Nanjing Jiancheng Bioengineering Institute, Nanjing, China) according to the manuals.

#### Blood Analysis

Blood gas including pH, PO_2_, TCO_2_, ${\text{HCO}}_{3}^{-}$, PCO_2_ and NH_3_ in sow plasma were analyzed using an i-STAT 1 blood gas system (Abbott Laboratories, Chicago, IL, USA). The serum uterine nitrogen (SUN) and total protein (TP) in sow serum were measured using commercially available kits (Nanjing Jiancheng Bioengineering Institute, Nanjing, China) according to the manual.

#### Fecal Urease Activity

Urease activity measurements in fresh feces were measured within 2 days using the indophenol blue colorimetry method described previously (13).

#### Fecal Ammonia Nitrogen Content

The ammonia nitrogen content of fresh feces was measured according to Chinese standard methods (HJ535-2009).

#### Total Nitrogen and Urea Nitrogen Content in Manure

Sow manure was made by mixing 60 g fresh feces and 60 ml of urine in a beaker. The fresh manure was then made homogenous by magnetic stirring at 300 rpm for 5 min. Approximately 6–8 g of manure were taken from the beaker to measure total nitrogen and urea nitrogen after 0, 12, 24, 48, 72, and 120 h of mixing (14). Total nitrogen was determined using the Kjeldahl method and urea nitrogen was determined using a commercial kit (Nanjing Jiancheng Bioengineering Institute, C013-2-2, Nanjing, China) according to the manual. Nitrogen loss during storage were calculated by total nitrogen at the beginning minus nitrogen content in the corresponding storage time.

### Data Analysis

All experimental data except for oestrus rate were analyzed by one-way analysis of variance (ANOVA) using SPSS 17.0 (SPSS, INC., Chicago, IL, USA) to determine whether significant variation existed among treatments. The least significant difference multiple range test was used to determine the differences between means when overall differences were found. The oestrus rate of sows was analyzed using a χ^2^ test. All data are shown as mean ± SEM except for oestrus rate, which was expressed in percentage. *P* < 0.05 was considered to be statistically significant and *P* < 0.01 was considered to be highly significant. Regarding sow and litter performance, the individual sow and her litter were used as the experimental unit.

## Results

### Animal Performance

There was no significant difference in live born piglets, viability of piglets, average birth weight, oestrus interval, and survival rate of the first piglet observed due to YSE treatment (Table 2). However, supplementation with YSE in late gestation diets resulted in a trend toward reduced number of stillbirth piglets (*P* = 0.08) and weak piglets (*P* = 0.06) compared to the basal diet (Tables 2, 3). The survival rate of the last three piglets was increased from 90.00% in the control group to 96.67% in the group with 0.06% YSE supplementation in the present study. Pre-weanling mortality were reduced from 7.78% in the control group to 2.86% in the groups with 0.06% YSE supplementation without changes in average daily gain (ADG) during lactation. In addition, the diarrhea rate of suckling piglets was significantly reduced in the groups with YSE supplementation compared with the control group (*P* = 0.03). The supplementation of YSE leas to a lower trend of diarrhea rated of weanling piglets (*P* = 0.06). Furthermore, the oestrus rate at day 5 and day 7 after weanling was effectively improved in all treatment groups with YSE supplementation.

**Table 2:** Effect of dietary YSE to sow reproduction performance.

**Items**	**Levels of YSE**				***P-*value**
	**0**	**0.06%**	**0.12%**	**0.24%**	
Parity live born piglets/litter	5.41 ± 0.51 10.23 ± 0.62	6.00 ± 0.49 10.00 ± 0.37	5.93 ± 0.54 10.33 ± 1.00	5.13 ± 0.41 10.31 ± 0.71	0.32 0.57
Stillbirth piglets/litter	0.61 ± 0.16	0.41 ± 0.21	0.40 ± 0.19	0.50 ± 0.20	0.08
Weak piglets/litter	0.22 ± 0.10	0.14 ± 0.11	0.13 ± 0.09	0.06 ± 0.06	0.06
Viability of piglets	3.55 ± 0.11	3.78 ± 0.13	3.61 ± 0.13	3.64 ± 0.18	0.24
Average birth weight (kg)	1.41 ± 0.24	1.39 ± 0.14	1.52 ± 0.26	1.45 ± 0.24	0.19
Birth litter weight (kg)	13.29 ± 1.65	13.95 ± 2.54	14.30 ± 3.00	14.77 ± 3.09	0.09
Oestrus interval (d)	4.89 ± 0.78	4.25 ± 0.62	4.51 ± 0.77	4.50 ± 0.67	0.16
Oestrus rate at D5 (%)	78.57 ± 9.19^a^	87.50 ± 8.92^b^	85.71 ± 7.35^b^	81.81 ± 7.19^ab^	0.04
Oestrus rate at D7 (%)	85.71 ± 7.24^a^	93.75 ± 8.39^b^	92.86 ± 9.77^b^	100^b^	0.04
Survival rate of the first piglet (%)	100	100	100	100	0.18
Survival rate of the last 3 piglets (%)	90.00 ± 3.18^a^	96.67 ± 2.08^b^	90.48 ± 4.14^a^	91.67 ± 2.11^a^	0.04

*D5, Day 5 after weanling; D7, Day 7 after weanling*.

*Weak piglets: birth weight < 1.1 kg*.

a,b *In a row, mean values with different superscript letters were significantly different (P < 0.05)*.

**Table 3:** Effect of dietary YSE to piglet performance.

**Items**	**Levels of YSE**				***P*-value**
	**0**	**0.06%**	**0.12%**	**0.24%**	
ADG (L2-7)	180.83 ± 9.04	174.30 ± 10.02	174.87 ± 15.40	184.24 ± 9.30	0.32
ADG (L7-15)	191.39 ± 10.39	197.06 ± 9.86	193.58 ± 9.01	195.70 ± 7.82	0.26
ADG (L15-21)	186.50 ± 10.41	189.16 ± 10.47	202.38 ± 11.04	193.13 ± 9.47	0.34
ADG (L2-15)	186.18 ± 7.53	185.69 ± 9.19	184.55 ± 9.78	189.97 ± 7.91	0.27
ADG (L2-21)	186.24 ± 6.45	188.55 ± 8.26	189.83 ± 9.37	191.59 ± 7.19	0.23
ADG (L21-31)	119.65 ± 8.22	117.43 ± 9.05	118.20 ± 8.95	121.06 ± 6.61	0.13
Mortality of piglets	7.78 ± 1.21^a^	2.86 ± 2.01^b^	6.91 ± 2.11^a^	6.25 ± 2.52^a^	0.04
Diarrhea rate of suckling piglets	9.16 ± 1.84^a^	3.64 ± 0.69^b^	3.17 ± 0.72^b^	4.77 ± 1.07^b^	0.03
Diarrhea rate of weanling piglets	6.58 ± 2.06	4.18 ± 1.18	6.73 ± 1.61	3.67 ± 0.67	0.06

*1) ADG, Average daily gain*.

a, b *2) In a row, mean values with different superscript letters were significantly different (P < 0.05)*.

*3) The incidence of diarrhea in piglets was observed and recorded 3 times per day during the study. Feces from each pig were scored by observe the shape and moisture content to assess the severity of diarrhea. Scores were 0, normal, firm feces; 1, possible slight diarrhea; 2, definitely unformed, moderately fluid feces; or 3, very watery and frothy diarrhea. diarrhea incidence (%) = number of pigs with diarrhea/(number of pigs × total experimental days) × 100*.

### Nutrient Apparent Digestibility

Apparent digestibility of dry matter was significantly higher in the sows fed 0.06 and 0.24% YSE supplement compared to those in the control group during gestation (*P* = 0.04). Additionally, the apparent digestibility of fat was elevated by 10% in the groups with 0.06% YSE supplement compared to the control group during lactation (*P* = 0.03). No obvious changes in protein digestion were observed (Table 4), but the urine nitrogen (UN) levels in sow manure during both late gestation and lactation were significantly suppressed with all levels of YSE supplementation included in present study (*P* = 0.03 and *P* = 0.02), while the total protein (TP) level was only significantly increased in those sows during late gestation and lactation (*P* = 0.02). There was a tendency to increase TP during late gestation in 0.24% treatment (*P* = 0.08) (Table 5). Among three supplementational levels, 0.06% YSE showed the best beneficial effect both on dry matter digestibility and fat digestibility.

**Table 4:** Effect of dietary YSE to nutrients apparent digestibility in gestating and lactating sow.

**Items (apparent digestibility)**	**Levels of YSE**				***P*-value**
	**0**	**0.06%**	**0.12%**	**0.24%**	
**D100 of gestation**					
Protein	83.89 ± 9.44	84.09 ± 8.43	84.19 ± 10.38	84.16 ± 6.17	0.23
Fat	63.56 ± 5.59	64.66 ± 6.63	63.60 ± 5.53	60.46 ± 5.54	0.14
Dry matter	81.23 ± 7.08^a^	87.64 ± 8.07^b^	86.48 ± 9.11^b^	82.58 ± 9.18^a^	0.04
**D15 of lactation**					
Protein	87.03 ± 0.54	88.22 ± 0.72	88.35 ± 0.78	87.11 ± 0.22	0.34
Fat	66.64 ± 7.97^a^	72.15 ± 6.04^b^	68.46 ± 7.91^ab^	68.53 ± 7.41^ab^	0.03
Dry matter	84.61 ± 0.43	84.92 ± 0.59	84.70 ± 0.74	84.72 ± 0.63	0.29

a,b *In a row, mean values with different superscript letters were significantly different (P < 0.05)*.

**Table 5:** The content of UN and TP in serum of sows with different levels of YSE supplementation.

**Period**	**Items**	**Levels of YSE**				***P*-value**
		**0**	**0.06%**	**0.12%**	**0.24%**	
G100	UN (mmol/L)	4.48 ± 2.20^a^	3.97 ± 2.30^b^	3.83 ± 1.19^b^	3.65 ± 3.14^b^	0.03
	TP (g/L)	66.15 ± 2.76	66.86 ± 4.31	67.07 ± 2.40	71.38 ± 1.62	0.08
L15	UN (mmol/L)	6.32 ± 4.61^a^	5.72 ± 3.24^ab^	4.96 ± 4.52^b^	4.81 ± 3.27^b^	0.02
	TP (g/L)	66.37 ± 5.58^A^	67.97 ± 4.38^A^	72.21 ± 6.77^B^	77.94 ± 8.24^B^	0.007

*1) UN, Urine nitrogen; TP, Total protein*.

a,b *2) In a row, mean values with different superscript letters were significantly different (P < 0.05)*.

### Blood Biochemical and Antioxidant Indices

No significant differences in sow blood oxyhemoglobin were induced by YSE supplementation during either gestation or lactation. Changes in other biochemical parameters, including pH, pCO_2_, pO_2_, ${\text{HCO}}_{3}^{-}$, TCO_2_, and NH_3_ (Table 6), induced by the supplementation of YSE, were not identified either. However, 0.12 and 0.24% YSE supplementation significantly enhanced sow blood catalase (CAT) activity during gestation and lactation, while malonaldehyde (MDA) levels during gestation were significantly lower in sows with 0.06% YSE supplementation (Table 7).

**Table 6:** Effect of dietary YSE to blood gas in gestating and lactating sow.

**Items**	**Levels of YSE**				***P*-value**
	**0**	**0.06%**	**0.12%**	**0.24%**	
**(A) D100 of gestation**					
Oxyhemoglobin (%)	76.70 ± 3.63	76.27 ± 3.72	73.23 ± 5.94	68.97 ± 5.50	0.14
pH	7.43 ± 5.03	7.43 ± 6.01	7.44 ± 7.01	7.43 ± 6.01	0.23
pCO_2_ (Kpa)	6.35 ± 6.11	6.23 ± 4.14	6.43 ± 5.13	6.43 ± 6.15	0.21
pO_2_ (Kpa)	5.71 ± 0.95	5.70 ± 1.44	5.34 ± 0.96	5.04 ± 0.98	0.32
${\text{HCO}}_{3}^{-}$(mmol/L)	32.16 ± 4.62	31.46 ± 3.48	32.83 ± 4.71	32.40 ± 4.25	0.15
TCO_2_ (mmol/L)	33.63 ± 4.63	32.90 ± 3.49	34.31 ± 4.73	33.87 ± 4.25	0.26
NH_3_ (mg/dL)	57.50 ± 6.23	63.44 ± 3.69	48.86 ± 6.23	54.17 ± 6.02	0.18
**(B) D15 of lactation**					
Oxyhemoglobin (%)	80.21 ± 7.12	82.92 ± 9.55	85.02 ± 7.46	80.44 ± 9.35	0.51
pH	7.42 ± 0.91	7.42 ± 0.81	7.42 ± 1.02	7.44 ± 1.01	0.22
pCO_2_ (Kpa)	6.05 ± 0.91	6.12 ± 0.94	6.04 ± 1.05	6.03 ± 0.84	0.34
pO_2_ (Kpa)	6.60 ± 0.94	6.46 ± 0.99	6.81 ± 0.81	6.46 ± 1.25	0.26
${\text{HCO}}_{3}^{-}$(mmol/L)	30.26 ± 4.63	29.97 ± 3.65	30.45 ± 4.81	30.94 ± 3.79	0.19
TCO_2_ (mmol/L)	31.69 ± 4.67	31.38 ± 3.67	31.83 ± 4.82	32.30 ± 5.81	0.28
NH_3_/(mg/dL)	25.88 ± 2.43	21.17 ± 4.42	30.50 ± 4.13	26.60 ± 5.20	0.31

*In a row, P < 0.05 means values were not significantly different*.

**Table 7:** Effect of dietary YSE to blood antioxidative parameter in gestating and lactating sow.

**Items**	**Levels of YSE**				***P-*value**
	**0**	**0.06%**	**0.12%**	**0.24%**	
**(A) D100 of gestation**					
T-AOC (U/mL)	6.93 ± 1.00	6.50 ± 0.42	7.11 ± 0.61	6.94 ± 0.75	0.14
GSH-PX (IU)	706.83 ± 37.36	791.30 ± 71.53	855.07 ± 80.69	804.35 ± 73.99	0.23
SOD (U/mL)	65.26 ± 1.39	61.18 ± 3.81	62.95 ± 1.85	63.40 ± 1.69	0.21
MDA (nmol/mL)	4.19 ± 0.29^a^	3.25 ± 0.11^b^	4.15 ± 0.21^a^	3.90 ± 0.89^ab^	0.04
CAT (U/mL)	1.52 ± 0.42^a^	1.74 ± 0.38^ab^	2.71 ± 0.41^b^	2.83 ± 0.46^b^	0.02
**(B) D15 of lactation**					
T-AOC (U/mL)	5.11 ± 0.83	6.66 ± 0.49	5.55 ± 0.66	6.58 ± 1.64	0.51
GSH-PX (IU)	627.82 ± 87.02	731.88 ± 96.39	739.13 ± 92.15	693.91 ± 61.94	0.22
SOD (U/mL)	64.51 ± 1.60	62.95 ± 0.80	61.18 ± 2.79	63.68 ± 1.77	0.34
MDA (nmol/mL)	3.89 ± 0.67	3.77 ± 0.26	3.55 ± 0.46	3.41 ± 0.38	0.26
CAT (U/mL)	1.85 ± 0.26^a^	1.70 ± 0.15^a^	3.01 ± 0.37^b^	2.97 ± 0.46^b^	0.04

*MDA, Malonaldehyde; CAT, Catalase; T-AOC, Total antioxidant capacity; SOD, Superoxide dismutase; GSH-Px, Glutathione peroxidase; GSH, Glutathione*.

a,b *In a row, mean values with different superscript letters were significantly different (P < 0.05)*.

### Total Nitrogen, Ammonia Nitrogen, Urea Nitrogen, and Urease Activity in Manure

**Figure 1:**
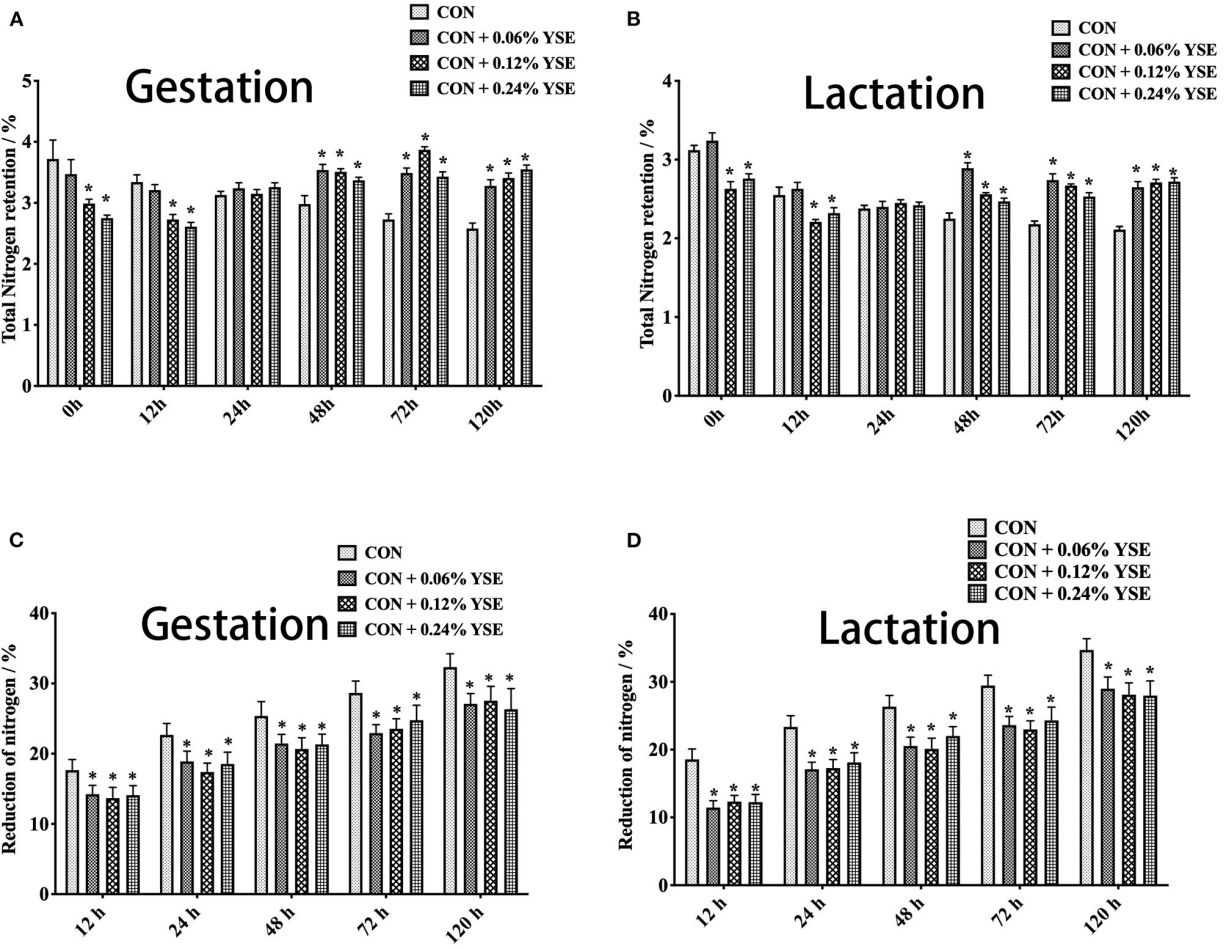
The effect of dietary YSE supplementation on total nitrogen content and loss of nitrogen in manure of gestating and lactating sow during storage. **(A)** The effect of dietary YSE supplementation on total nitrogen content in manure of gestating sows during storage; **(B)** The effect of dietary YSE supplementation on total nitrogen content in manure of lactating sows during storage; **(C)** The effect of dietary YSE supplementation on loss of nitrogen in manure of gestating sows during storage; **(D)** The effect of dietary YSE supplementation on loss of nitrogen in manure of lactating sows during storage. *means significant difference.

**Figure 2:**
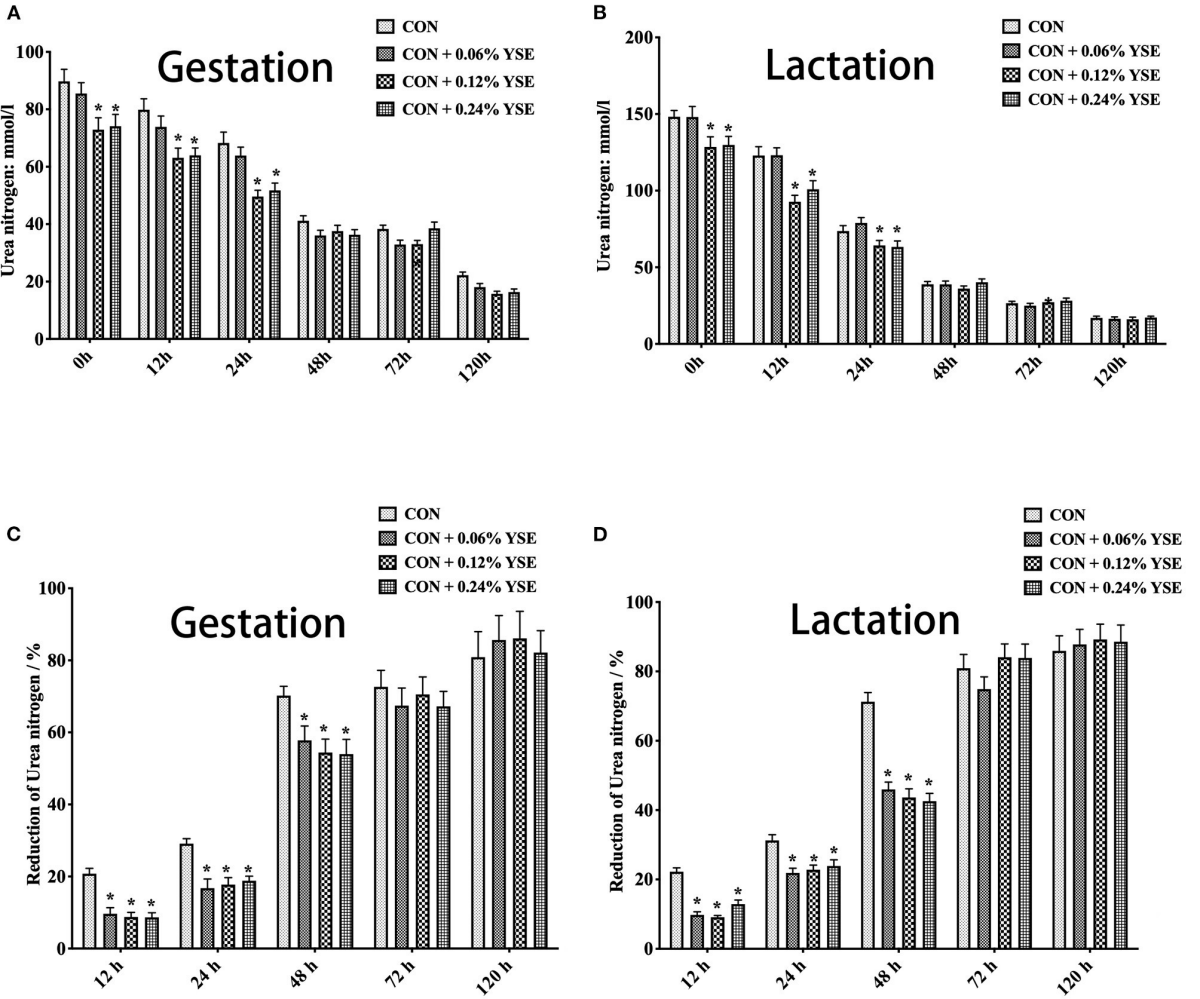
The effect of dietary YSE supplementation on urea nitrogen content and loss of urea nitrogen in manure of gestating and lactating sows during storage. **(A)** The effect of dietary YSE supplementation on urea nitrogen content in manure of gestating sows during storage; **(B)** The effect of dietary YSE supplementation on urea nitrogen content in manure of lactating sows during storage; **(C)** The effect of dietary YSE supplementation on loss of urea nitrogen in manure of gestating sows during storage; **(D)** The effect of dietary YSE supplementation on loss of urea nitrogen in manure of lactating sows during storage. *means significant difference.

The results showed that initial total nitrogen contents and urea nitrogen in manure at 0 h of storage were significantly lower in those groups with 0.12 and 0.24% supplementation of YSE during gestation and lactation (Figures 1A,B, 2A,B). Besides, total nitrogen content in manure from gestating and lactating sows fed with 0.12 and 0.24% supplemental YSE continuously decreased at 12 h of storage, but this trend disappeared at 24 h and then reversed at 48 h and until to 120 h (Figures 1A,B). The total nitrogen contents in manure of both gestation and lactation was significantly higher in those sows supplemented with YSE compared with control sows during 48–120 h of storage (Figures 1A,B).

We further calculated the nitrogen loss of manure during storage and, interestingly found that all groups with YSE supplementation had less nitrogen loss during the entire storage period (Figures 1C,D). The loss of urea nitrogen during storage in the groups with YSE supplementation showed significantly lower values compared with the control group during gestation and lactation (Figures 2C,D), with the results being consistent with the trend of total nitrogen content.

**Figure 3:**
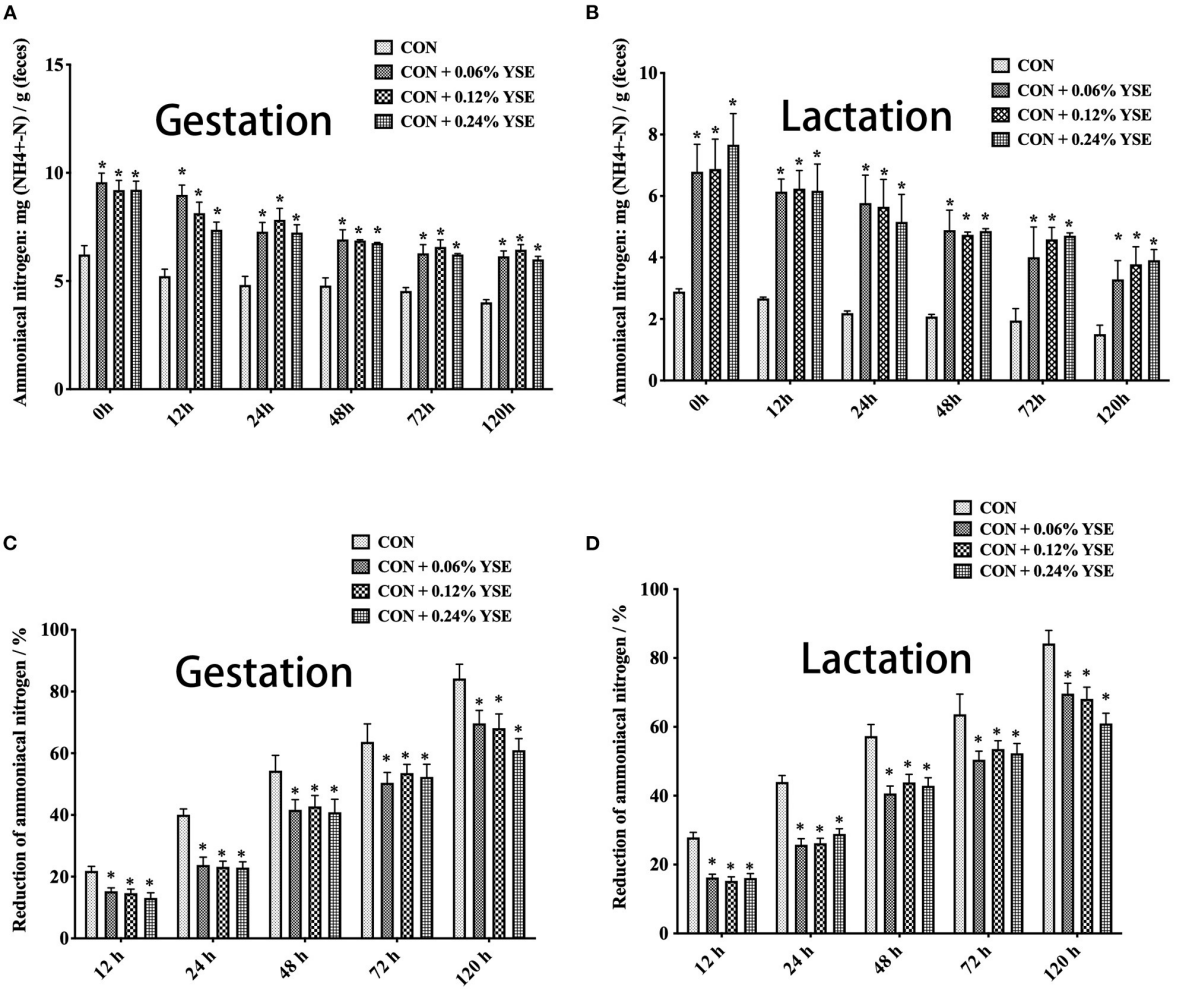
The effect of dietary YSE supplementation on ammoniacal nitrogen content and loss of ammoniacal nitrogen in manure of gestating and lactating sows during storage. **(A)** The effect of dietary YSE supplementation on ammoniacal nitrogen content in manure of gestating sows during storage; **(B)** The effect of dietary YSE supplementation on ammoniacal nitrogen content in manure of lactating sows during storage; **(C)** The effect of dietary YSE supplementation on loss of ammoniacal nitrogen in manure of gestating sows during storage; **(D)** The effect of dietary YSE supplementation on loss of ammoniacal nitrogen in manure of lactating sows during storage. *means significant difference.

**Figure 4:**
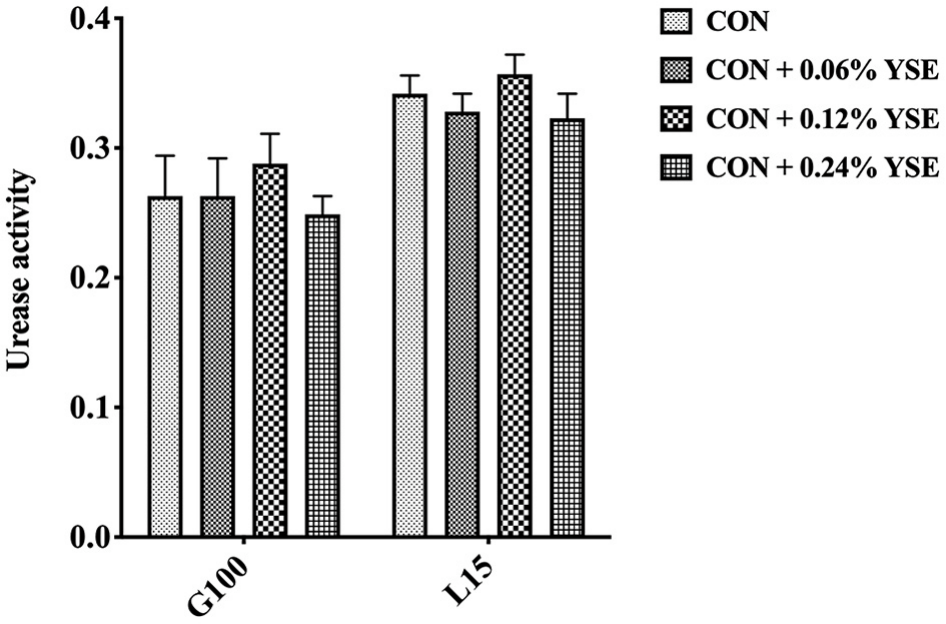
The effect of YSE supplementation on urease activity in sow manure during gestation and lactation.

The contents of ammonia nitrogen in manure from those lactating and gestating sows supplemented with YSE were significantly higher during the entire storage compared with the control group (Figures 3A,B). However, the loss of ammonia nitrogen in manure was significantly less than in those groups with YSE supplementation during both gestation and lactation (Figures 3C,D). We did not observe a significant effect of YSE supplementation on urease activity in manure during gestation or lactation (Figure 4).

## Discussion

### Animal Performance

The positive effects of dietary supplementation of YSE on growth rate, feed efficiency, and animal health during the grower period has been reported in previous studies (15–18). However, there have been limited investigations focusing on the sow production. In the present study, we evaluated the effect of YSE supplementation on sow and litter performance from late pregnancy (G100) to lactation (L21), with the results shown in Tables 2, 3. The results of our study are in accordance with the report of Cline, who either did not find a beneficial effect of supplementing YSE in sow diets on prenatal growth of piglets, but observed significant reduced stillbirth occurrence (41.2%) (19). Herpin et al. also reported that the number and percentage of stillbirths per litter were reduced from 0.78 and 7.2% to 0.5 and 4.7%, respectively when YSE powder was supplemented into the gestational sow diet (20). In addition, increased survival rate of the last three piglets and decreased piglet mortality and diarrhea rate in present study further confirmed the beneficial effect of YSE on sow performance and postnatal growth in piglets. We observed that all levels of YSE supplemetantion significantly improved diarrhea rate of suckling piglets but no obviouse affect on diarrhea rate of weaning piglets (Table 3). This result might because that YSE could improve piglets diarrhea rate by increasing antioxidative capacity, but oxidative stress during weanling was too extensive to eliminate only by supplementation of YSE, which resulting in no significant change in diarrhea rate of weanling pielets. It is interesting that this benefit disappeared when the supplementation were increased up to 0.12% and 0.24% indicating 0.06% supplementaion would be the optimal dose for animal performance during late gestataion and lactation. Yucca is a source of steroid saponins that have phyto-estrogenic effects through binding to estrogen receptors and consequently affect those processes regulated by estrogen (21–23). Several previous studies reported that addition of yucca in feed positively affects ovarian function and influences the reproductive process in cattle, rabbits, pig and goat (24–26). In the present study, the increased oestrus rate at day 5 and day 7 with YSE supplementation might be due to the fact that the steroid saponins contained in YSE have a certain promoting effect on the recovery of ovarian and uterus after pregnancy (27).

We also determined the effect of YSE supplementation on nutrient apparent digestibility and the results with higher dry matter and fat digestibly are consistent with the previous study conducted by Min et al., who reported addition of yucca extracts to the diet showed significantly higher dry matter, crude ash and crude protein digestibility (28). Lila et al. also showed beneficial effects of YSE on ruminal ammonia levels, DM digestibility and rumen pH in lactating cows (29). The beneficial effect of YSE on nutrient utilization might be attributed to the characteristic properties of saponins, the main active components in yucca extract, to promote fat emulsification due to its surface-activity and the capacity to delay the passage of intestinal chyme (30).

### Blood Biochemical and Antioxidative Indices

Deficiency in blood oxygen supply has been considered to be a crucial factor in fetal and piglet death during gestation, parturition and lactation (31, 32). Our results showed clearly that supplementation of YSE effectively decreased fetal and piglet mortality during the entire reproductive cycle. Cline showed that supplementation of YSE to sows prior to the start of farrowing resulted in increased blood oxygen supply to the fetus during parturition (19), which could be the reason for fewer stillbirths and decreased pre-weaning mortality. However, Heprin et al. observed no obvious changes in oxygen content either in the umbilical cord vein at birth or in the umbilical artery at 1 h after birth with the supplementation of YSE, and they proposed that beneficial effects of YSE on blood oxygen content were only significant in sub-optimal oxygenated conditions (20). In our present study, no significant differences in sow blood oxyhemoglobin were induced by YSE supplementation during either gestation or lactation. The different oxygenated conditions, which were the result of variations in the rearing environment and feed composition, might be the reason for the contradictory results in these studies remaining to be addressed by future investigations.

A number of previous studies have reported that YSE can effectively relieve oxidative damage in various species, including the rat, cattle, and sheep (33–35). However, there have been few studies on the effect of YSE on antioxidative function in pig, especially in sow. In the study described herein, we determined blood antioxidant parameters to evaluate the protective role of dietary supplementation of YSE against basal oxidative damage, both in sows and piglets. The results showed that YSE supplementation significantly enhanced sow antioxidative status and these observations were consistent with previous studies in other species. It has been suggested that the benefit of YSE as a natural antioxidant can be attributed to the phenolic hydroxyl groups included in yucca, which serve as hydrogen donors to the peroxy radicals produced by lipid oxidation, thus suppressing the formation of hydroxyl peroxide (33, 36).

### Total Nitrogen, Ammonia Nitrogen, Urea Nitrogen, and Urease Activity in Manure

Nitrogen in animal manure, including urea nitrogen and undigested protein from feed, could be hydrolyzed by fecal microbial ureases to be ammonia and then volatilized to the atmosphere, which results in loss of nitrogen in manure and increase in ammonia release during storage (37). YSE has been reported to reduce ammonia emission from animal manure in several previous studies. Piacente et al. showed the positive effects of dietary YSE supplementation to different animals on ammonia levels in air (38). Santacruz-Reyes and Chien reported that the application of YSE in water or feed could reduce the accumulation of ammonia in wastewater discharge during shrimp production (39). Saeed et al. (9) reported that YSE could mitigate ammonia emissions from poultry manure and promoted animal health and production. In present study, we tracked the temporal change of total nitrogen and urea nitrogen in sow manure during storage and found that 0.12 and 0.24% YSE inclusion in sow diet could improve protein utilization in both gestating and lactating sows (Figures 1A,B, 2A,B), which result were consistent with our results about the effect of YSE to nutrients digestation in present study. The change of total nitrogen contents and urea nitrogen at different time point of storage might be due to higher microbial degradation rate at the begin of storage because of more abundant nutrients when manure was relatively fresh, and then microbial degradation rate decreased with time because of exhaustion of the nutrient supply by microbes (40). Although the level of 0.06% YSE supplementation were too low to lead to significant change on total nitrogen and urea nitrogen content of sow manure, all levels of supplementation of YSE could greatly decreased the reduction of total nitrogen and urea nitrogen of sow manure, implying the beneficial effect of YSE addition to reduce ammonia emissions of sow manure during storage.

The beneficial effect of YSE to control ammonia emission from animal manure has been observed in previous and our present studies, there is still much controversy surrounding the mechanism by which YSE has a positive effect in reducing ammonia volatilization. Makkar et al. and Cunningham and Morrison reported that YSE may efficiently entrap ammonia nitrogen in wheat straw ingested into the rumen of the cow, a potentially advantageous situation as it could lead to lower emission of polluting gases (41–43). Headon and Dawson and Wu et al. also pointed out that YSE has ammonia-binding properties due to the presence of a glycoprotein (44, 45), which plays an important role in reducing ammonia output from animal excretions. Inconsistently, other reports indicate that the beneficial effect of YSE on ammonia emission reduction should be attributed to the fact that its saponin fractions inhibit the activity of urease on the decomposition of urea nitrogen to be ammonia (46, 47).

The conversion from organic nitrogenous materials to ammonia is an enzymatic reaction, in which urease plays an important role as the limiting enzyme for the last step and ammonia nitrogen is included as key intermediate product (48, 49). The higher content of ammonia nitrogen in manure would lead to suppression of NH_3_ release. Thus, with the intent to answer the question as to how YSE reduces the emission of ammonia, we determined urease activity, ammonia nitrogen, and loss of ammonia nitrogen in manure at different time points of storage. The results showed that ammonia nitrogen content in sows feces supplemented with all levels of YSE included in previous study during lactation and pregnancy was significantly higher than that in the control group throughout the storage process (Figures 3A,B), while the loss of ammonia nitrogen in manure was significantly less than in those groups with YSE supplementation during both gestation and lactation (Figures 3C,D), which implying more ammonia nitrogen were kept in manure and could not be convert to ammonia to emit into air. Combining to the observation that no significant change of urease activity in manure in the group with YSE supplementation, which results are also consistent with the *in vivo* or *in vitro* study's of Killeen et al. (50, 51), we speculate that YSE might entrap ammonia nitrogen in manure to reduce ammonia emission during storage.

## Conclusion

In summary, results from this study indicate that 0.06% supplementation of YSE in the sow diet during gestation and lactation could improve sow reproductive performance with increased survival rate of the last three piglets, as well as reduced pre-weanling mortality and diarrhea rate in the piglets. Besides, 0.06, 0.12, and 0.24% YSE supplementation could reduce loss of total nitrogen and urea nitrogen in sow manure, while decreasing ammonia emission by binding ammonia nitrogen and inhibiting its degradation. The results of this study provided practical information about application of YSE (optimal supplementation level for different physiological stages) in sow diet to improve reproductive performance and reduce ammino emission in pig industry. Further investigation are needed to performed to study the effect of YSE to milk quality which might be the crucial reason for reduced mortality rate of piglets in present study.

## Data Availability Statement

The raw data supporting the conclusions of this article will be made available by the authors without reservation.

## Ethics Statement

The animal study was reviewed and approved by South China Agricultural University.

## Author Contributions

Material preparation, data collection, and analysis were performed by CW, PZ, and CC. The first draft of the manuscript was written by FC and WG. All authors contributed to the study conception and design, commented on previous versions of the manuscript, read, and approved the final manuscript.

## Conflict of Interest

The authors declare that the research was conducted in the absence of any commercial or financial relationships that could be construed as a potential conflict of interest.

## Abbreviations

YSE: *Yucca schidigera* extract
ADG: Average daily gain
MDA: Malonaldehyde
CAT: Catalase
T-AOC: Total antioxidant capacity
SOD: Superoxide dismutase
GSH-Pxm: Glutathione peroxidase
GSH: Glutathione
UN: Urine nitrogen
TP: Total protein.
